# Haploinsufficient TNAP Mice Display Decreased Extracellular ATP Levels and Expression of Pannexin-1 Channels

**DOI:** 10.3389/fphar.2018.00170

**Published:** 2018-03-02

**Authors:** Álvaro Sebastián-Serrano, Laura de Diego-García, David C. Henshall, Tobías Engel, Miguel Díaz-Hernández

**Affiliations:** ^1^Department of Biochemistry and Molecular Biology, Veterinary School, Complutense University of Madrid, Madrid, Spain; ^2^Instituto de Investigación Sanitaria del Hospital Clínico San Carlos, Madrid, Spain; ^3^Department of Physiology and Medical Physics, Royal College of Surgeons in Ireland, Dublin, Ireland; ^4^FutureNeuro Research Centre, Dublin, Ireland

**Keywords:** tissue-nonspecific alkaline phosphatase (TNAP), adenosine 5′-triphosphate (ATP), hypophosphatasia (HPP), P2X7 receptor (P2X7R), pannexin 1 (Panx1), seizure, epilepsy, connexins

## Abstract

Hypophosphatasia (HPP) is a rare heritable metabolic bone disease caused by hypomorphic mutations in the *ALPL* (in human) or *Akp2* (in mouse) gene, encoding the tissue-nonspecific alkaline phosphatase (TNAP) enzyme. In addition to skeletal and dental malformations, severe forms of HPP are also characterized by the presence of spontaneous seizures. Initially, these seizures were attributed to an impairment of GABAergic neurotransmission caused by altered vitamin B6 metabolism. However, recent work by our group using knockout mice null for TNAP (TNAP-/-), a well-described model of infantile HPP, has revealed a deregulation of purinergic signaling contributing to the seizure phenotype. In the present study, we report that adult heterozygous (TNAP+/-) transgenic mice with decreased TNAP activity in the brain are more susceptible to adenosine 5′-triphosphate (ATP)-induced seizures. Interestingly, when we analyzed the extracellular levels of ATP in the cerebrospinal fluid, we found that TNAP+/- mice present lower levels than control mice. To elucidate the underlying mechanism, we evaluated the expression levels of other ectonucleotidases, as well as different proteins involved in ATP release, such as pannexin, connexins, and vesicular nucleotide transporter. Among these, Pannexin-1 (Panx1) was the only one showing diminished levels in the brains of TNAP+/- mice. Altogether, these findings suggest that a physiological regulation of extracellular ATP levels and Panx1 changes may compensate for the reduced TNAP activity in this model of HPP.

## Introduction

Intracellular ATP, best known as the main source of chemical energy along with its involvement in many crucial cellular processes, is also widely recognized to act as either sole transmitter or co-transmitter in both the peripheral and CNS (Burnstock, 2013; Rodrigues et al., 2015). In the CNS, the extracellular concentration of ATP is tightly regulated under physiological conditions via different mechanism controlling its release and degradation (Burnstock, 2016). ATP can reach the cerebral parenchyma through exocytotic and conductive release (Lazarowski et al., 2011). Exocytosis of ATP requires prior storage of ATP in secretory vesicles via VNUT (Sawada et al., 2008). Additional mechanisms of nucleotide release include ATP-binding cassette transporters, connexin or pannexin hemichannels (Dubyak, 2006; Scemes et al., 2007). During pathological conditions, however, rupture of the plasma membrane because of cell death or damage causes intracellular ATP to be directly released, thereby rapidly increasing extracellular ATP levels (Rodrigues et al., 2015).

In the extracellular space, ATP and other nucleotides undergo rapid hydrolysis by enzymes called ectonucleotidases. These enzymes are found at the plasma membrane and possess an extracellularly oriented catalytic site. This enzyme family includes ecto-nucleoside triphosphate diphosphohydrolases (E-NTPDases), ecto-nucleotide pyrophosphatase/phosphodiesterases (E-NPPs), alkaline phosphatases and ecto-5′-nucleotidase (Zimmermann, 2006). Regarding alkaline phosphatases, their mammalian isoforms share an optimum alkaline pH and are anchored to the membrane via a glycosylphosphatidylinositol anchor (Millan, 2006). TNAP is expressed in a multitude of tissues including liver, bone, kidney, and brain. In the adult mammalian CNS, TNAP represents the only isoform of alkaline phosphatases and is associated with the blood vessel endothelium and with neuropil, including synaptic contacts (Langer et al., 2008). Hypophosphatasia is a rare, inborn-error-of-metabolism characterized by defective mineralization of bone and/or teeth caused by loss-of-function mutations in the gene encoding TNAP (*ALPL* in humans and *Akp2* or *Alpl* in mice) (Whyte et al., 2015). Clinical symptoms differ with age of onset, with the perinatal form being the most severe. Neonates affected by HPP suffer from impairment of bone mineralization, respiratory distress, and spontaneous seizures which ultimately lead to death within weeks after birth (Whyte et al., 2015).

Initial studies using *Akp2* knockout (TNAP-/-) mice, a well-establish model of infantile HPP, suggested that seizures were a consequence of diminished levels of GABA in the brain, caused in turn by a defective metabolism of vitamin B6 (Waymire et al., 1995; Narisawa et al., 1997, 2001). However, our group recently identified additional phenotypes in TNAP-/- mice including increased proliferation of neural precursors, altered neuronal morphology, and augmented neuronal activity (Sebastián-Serrano et al., 2016). These morphological alterations were found to result from persistent activation of the purinergic P2X7 receptor (P2X7R), caused by the high concentrations of extracellular ATP derived from a deficient activity of TNAP. Moreover, we demonstrated that exogenous ATP or TNAP antagonists were able to trigger seizures in adult mice, with heterozygous TNAP (TNAP+/-) mice being more sensitive to ATP-induced seizures than WT mice. Accordingly, the blockage of P2X7R prevented seizure occurrence in the HPP mouse model (Sebastián-Serrano et al., 2016).

Current enzyme replacement therapy has shown skeletal, respiratory, and functional improvements as well as prevention of seizures in the most severe perinatal cases. However, several adverse side effects such as vascular calcification which has been described as comorbidity of aging, diabetes mellitus, or chronic kidney disease have been reported (Millan and Whyte, 2016). Hence, new alternative therapeutic strategies independent on ALPs targeting are currently explored. Based on our previous results, in the present study we tried to determine whether factors regulating extracellular ATP levels can be considered as potential therapeutic targets for HPP via avoiding the pathological increase of extracellular ATP concentration caused by a deficiency in TNAP activity. To address this point, we decided to use TNAP+/- mice for several reasons; although they present a decreased genetic load of alkaline phosphatase, they do not develop spontaneous seizures (Waymire et al., 1995; Narisawa et al., 1997), they are more sensitive to ATP-induced seizures than WT mice (Sebastián-Serrano et al., 2016) and they have a higher life expectancy than TNAP-/- mice, who die around postnatal day 10. Using this mouse model, we measured CSF levels of ATP, studied possible ecto-ATPase activity compensations by other ectonucleotidases and finally, focused on some of the main proteins implicated in the extracellular release of ATP.

## Materials and Methods

### Animals

All animal procedures were carried out at the Complutense University of Madrid, in compliance with National and European regulations (RD1201/2005; 86/609/CEE) following the guidelines of the International Council for the Laboratory Animal Science. The protocol was approved by the Committee of Animal Experiments of the Complutense University of Madrid and the Environmental Counseling of the Comunidad de Madrid, Spain. All surgery was performed under isoflurane anesthesia, and all efforts were made to minimize suffering.

TNAP-/- mice were generated by the inactivation of the mouse *Akp2* gene, as previously described (Narisawa et al., 1997) and generously provided by Prof. Jose L. Millán (Sanford Burnham Medical Research Institute, La Jolla, CA, United States). WT, TNAP+/- and TNAP/- mice came from heterozygous TNAP+/- breeding pairs and were housed with food and water available *ad libitum* and maintained in a temperature-controlled environment on a 12/12 h light/dark cycle with light onset at 08:00 A.M.

### PCR Genotyping

Genomic DNA was obtained from tail biopsies using Wizard^®^ SV Genomic DNA Purification System (Promega, Madison, WI, United States) according to the manufacturer’s protocol.

Simple PCR reactions were carried out using DNA Amplitools Master Mix (Biotools, Madrid, Spain), specific primers (400 nM each) and 5 μL of genomic DNA in a final volume of 25 μL. Animals were genotyped using specific primers for TNAP Forward 5′-TGCTGCTCCACTCACGTCGAT-3′ and Reverse 5′-AGTCCGTGGGCATTGTGACTA-3′. PCR was carried out over 40 cycles of 94°C for 30 s, 58°C for 1 min, and 72°C for 5 min.

PCR amplification products were electrophoresed on a 1.5% (w/v) agarose gel and stained with SYBR^®^ Safe DNA Gel Stain (Life Technologies, Foster City, CA, United States). PCR bands were visualized by gel imaging system Gel Logic 200 Imaging System (Kodak, Rochester, NY, United States).

### Tissue Processing for Immunohistochemistry

Adult animals were anesthetized using a mix of ketamine (80–200 mg/kg) and xylazine (7–20 mg/kg) diluted in PBS (137 mM NaCl, 2.7 mM KCl, 5 mM Na_2_HPO_4_ 7H_2_O, 1.4 mM KH_2_PO_4_; pH 7.4) and administered as a single intraperitoneal injection. Mice were perfused transcardially with PBS followed by cold PFA (pH 7.4) (Sigma-Aldrich). Perfused brains were dissected immediately and placed overnight in 4% PFA at 4°C for post-fixation. Excess PFA was removed with three PBS washes. Next, fixed brains were placed in 30% sucrose in PBS overnight at 4°C for cryoprotection. Then samples were embedded in OCT compound (Sakura) and frozen using dry ice. Finally, 30 or 50 μm floating sections were cut in parasagittal planes with a cryostat (CM1950, Leica Microsystems) and stored in a solution of 30% ethylene glycol, 30% glycerol and 0.1 M PBS at -20°C until processed.

### Enzyme Histochemistry

*Alkaline phosphatase activity* on brain slices from adult WT and TNAP+/- mice was detected using BCIP/NBT (0.35 mM BCIP, 0.37 mM NBT, 5 mM MgCl_2_, 100 mM Tris buffer, pH 9.5) as a dark precipitating substrate according to the manufacturer’s protocol (Sigma-Aldrich, St. Louis, MO, United States). Briefly, free-floating sections were washed twice with PBS and rinsed with Tris-HCl buffer (pH 7.5) and incubated with substrate about 40 min until optimal staining intensity was obtained. After washing with Tris-HCl buffer, sections were mounted on glass slides with an anti-fading solution. To quantify the enzymatic reaction, we measured the amount of BCIP/NBT precipitate deposited on the hippocampus of WT and TNAP+/- mice. Values correspond to the mean intensity value of the pixel in eight bytes images containing the hippocampus (0–255 scale with 0 = white and 255 = black).

*ATPase activity* was visualized as previously described (Gampe et al., 2015). Briefly, brains sections from adult WT and TNAP+/- mice were washed in PBS and pre-treated with TMS (0.25 M sucrose, 50 mM Tris-maleate, 2 mM MgCl_2_, pH 7.4) for 30 min at room temperature. The enzymatic reaction was carried out in TMS-buffered substrate solution stabilized with 3% dextran T 40 (Roth) [TMS-S; 2 mM Pb(NO_3_)_2_, 5 mM MnCl_2_, 2 mM MgCl_2_, 50 mM Tris-maleate, pH 7.4, plus 0.25 M sucrose] containing the substrate 1 mM ATP (Sigma-Aldrich) for 90 min at 37°C. The lead the dark orthophosphate precipitated, as a result of nucleotidase activity, was visualized as a brown deposit after to incubate sections for 1 min in an aqueous solution of (NH_4_)_2_S (1% v/v) (Sigma-Aldrich). Subsequently, sections were washed twice with PBS and mounted on glass slides with an anti-fading solution. To quantify the enzymatic reaction, we measured the amount of orthophosphate precipitate deposited on the hippocampus of WT and TNAP+/- mice. Values correspond to the mean intensity value of the pixel in eight bytes images containing the hippocampus (0–255 scale with 0 = white and 255 = black).

### Immunohistochemistry Techniques

Fresh-frozen parasagittal brain sections from adult WT and TNAP+/- mice were stained free-floating using the biotin–avidin–peroxidase method, with DAB as a chromogen. Endogenous peroxidase was inactivated by incubating sections in a solution of 0.3% hydrogen peroxide in PBS for 30 min. Brain sections were washed in PBS, blocked for 1 h at room temperature with 1% BSA, 5% FBS, and 0.2% Triton X-100 (Sigma-Aldrich) in PBS, and subsequently incubated with rabbit polyclonal antibody against Caspase 3 (catalog 9661, Cell Signaling, 1:50). Finally, brain sections were incubated with avidin–biotin complex using Elite Vectastain kit (Vector Laboratories). Chromogen reactions were performed with DAB (SigmaFAST DAB, Sigma-Aldrich) and 0.003% H_2_O_2_ for 10 min. Once washed, sections were mounted on glass slides and after dried were coverslipped using FluorSave (Calbiochem).

### Image Acquisition

Transmitted light images were acquired using a microscope (Eclipse TE200, Nikon) with DFC310FX camera (Leica Microsystems GmbH) using Leica Application Suite (v4.1). Sections were photographed with Plan 4× dry objective lens (NA = 0.1) and insets with Plan S-Fluor 40× dry objective lens (NA = 0.90, Nikon) at room temperature.

### Quantitative Real-Time PCR (qRT-PCR)

Total RNA was extracted from hippocampi of adult mouse brains using a Speedtools total RNA Extraction Kit (Biotools). Briefly, animals were sacrificed by cervical dislocation and the hippocampi were immediately dissected and frozen using dry ice to procedure with total RNA isolation according to the manufacturer’s instruction. After digestion with TURBO DNase (Ambion), 1 μg of total RNA was reverse transcribed with 6 μg of random primers, 350 μM dNTPs and M-MLV reverse transcriptase (all from Invitrogen). qRT-PCR reaction mixtures containing DNA Master SYBR Green I mix (Applied Biosystems) were incubated at 95°C for 20 s followed by 40 PCR cycles (95°C for 1 s and 60°C for 20 s) in a StepOnePlus Real-Time PCR System (Applied Biosystems). Specific primers for *Entpd1* were 5′-AAAGCCATGCAGTGCCTTTG-3′, 5′-GCAAGGACTCTTTGGCTTTAGC-3′; for *Entpd2* were 5′-AGCTACGCAAATGACCCTTC-3′; 5′-TGGAGTGCTGGCATATCTGTC-3′; for *Entpd3* were 5′-AGGTGGCTTCTGTGTTTGAC-3′, 5′-AACCTTGGGCTGGTAAATGC-3′; for *Enpp1* were 5′-AAAGGCCGCTGCTTTGAAAG-3′, 5′-ACCGCACCTGAATTTGTTGC-3′; for *Enpp2* were 5′-ACCTTCCCAAACGTTTGCAC-3′, 5′-AGGTTTCCTTGCAACATGCC-3′; for *Enpp3* were 5′-ATGACGTGCACCTCAACAAG-3′, 5′-ATTGCCGTTAGCCAAATCGG-3′. Expression levels of mRNA were represented as 2^-Δ ΔCt^, where average cycle threshold (Ct) was obtained from triplicates of each sample. First, ΔCt means normalization to parallel amplification of GAPDH as endogenous control. Next, ΔΔCt means normalization to the average of corresponding controls.

### *In Vivo* Seizure Induction and Recording

First, WT or TNAP+/- mice were anesthetized using isoflurane (3–5%) and maintained normothermic by means of a feedback controlled heat blanket (Harvard Apparatus, Ltd., Kent, United Kingdom). Mice were then placed in a stereotaxic frame, and three partial craniotomies were performed to affix cortical skull-mounted EEG electrodes (Bilaney Consultants, Ltd., Sevenoaks, United Kingdom). EEG was recorded using a Grass Comet XL digital EEG (Medivent, Ltd., Lucan, Ireland) at band-width 1–70 Hz (Notch filter was set to 50 Hz) and Sampling rate +800 Hz. A guide cannula was affixed (coordinates from Bregma: AP = 0.4 mm; L = 0.95 mm) and the entire skull assembly was fixed in place with dental cement. Baseline EEG was recorded for at least 10 min, and then an injection cannula was lowered through the guide cannula for i.c.v. injection of 2 μL vehicle or 0.5 M ATP at 10 s/μL (Sigma-Aldrich). Mice were monitored up to 2 h after ATP injection. Automated EEG frequency analysis was performed by uploading EEG into Labchart7 software (ADInstruments, Ltd., Oxford, United Kingdom).

### CSF Acquisition and ATP Measurement

Briefly, mice were anesthetized with isoflurane diluted in a 50% O_2_ and placed in a stereotaxic frame with the head forming a nearly 135° angle with the body. Mice were kept under anesthesia during the surgery. The CSF of WT and TNAP+/- mice was collected from the cisterna magna with a pulled capillary. Blood vessels were carefully avoided when penetrating the dura mater with the capillary tube to prevent contamination from plasma proteins. CSF was placed in a tube with 100 μM ARL 67156, a competitive inhibitor of ecto-ATPases (Levesque et al., 2007) and immediately frozen on dry ice to further determine the ATP concentration.

The nucleotide concentration in the CSF was measured using ENLITEN^®^ rLuciferase/Luciferin reagent (Promega) according to the manufacturer’s protocol. Briefly, 1 μL CSF from WT or TNAP+/- mice was transferred to wells of a 96-well plate placed on ice. The 96-well plate was set in a FLUOstar OPTIMA Microplate Luminometer (BMG LABTECH GmbH), and 100 μL of rLuciferase/Luciferin reagent was automatically injected into each well at room temperature.

### Western Blot

Total protein extracts from hippocampus and cortex of adult and neonatal mouse brains were lysed and homogenized mechanically for 1 h at 4°C in lysis buffer containing 20 mM Hepes, 100 mM NaCl, 50 mM NaF, 5 mM EDTA, 5 mM Na_3_VO_4_ (all salts from Sigma-Aldrich), 1% Triton X-100, okadaic acid (Calbiochem), and Complete TM Protease Inhibitor Cocktail Tablets (Roche Diagnostics GmbH), pH 7.4. Protein quantity was detected using Bradford Protein Assay (Bio-Rad), and equal amounts of protein were used for Western blot analysis. Separation of the proteins was performed on 10 or 12% SDS-PAGE gels. Immunotransference was carried out in nitrocellulose membranes (Amersham Biosciences). The PBS-buffer containing 0.1% (v/v) Tween-20 (PBS-T) and 5% non-fat dried milk was used as a blocking medium for 1 h at room temperature. Incubation with antibodies was performed overnight at 4°C at the dilutions specified in parentheses: goat anti-Pannexin-1 antibody (catalog SC49695, Santa Cruz Biotechnology, 1:100), mouse anti-connexin 32 antibody (catalog 13-8200, Life Technologies, 1:100), mouse anti-connexin 43 antibody (catalog 13-8300, Life Technologies, 1:100), rabbit anti-VNUT (catalog PA5-63312, Thermo Fisher Scientific, 1:50), rabbit anti-VNUT (catalog ABN110; Millipore, Billerica, MA, United States 1:500) or rabbit anti-GAPDH (catalog G9545, Sigma-Aldrich, 1:40.000). The membranes were washed for 10 min with PBS-T three times, and incubated with secondary antibodies rabbit anti-goat, goat anti-rabbit or anti-mouse IgGs coupled to horse-radish peroxidase (HRP, Amersham GE Healthcare) for 1 h at room temperature used at 1:1000, 1:1000, or 1:5000, respectively. Protein bands were visualized by ECL Pro chemiluminescence (Amersham GE Healthcare). Protein expression was standardized by the expression of GAPDH from the same experiment. Gel band images densities were captured using ImageQuant LAS 500 (GE Healthcare Life Sciences) and analyzed using ImageJ software (v1.47d, NIH, Bethesda, MD, United States), without applying any background subtraction. In the figures, the representative Western blot images show only the quantified bands. Full size images are presented in Supplementary Figure 5.

### Immunofluorescence Studies

For confocal microscopy, animals were transcardially perfused with 4% PFA for 10 min, post-fixed, and cryoprotected in 30% sucrose before sectioning. Tissue slices were washed in PBS and treated with blocking solution containing 5% FBS, 1% BSA, and 0.2% Triton X-100 in PBS buffer. After that, samples were incubated with primary antibodies diluted in blocking solution. After washing, sections were incubated with fluorescent-tagged secondary antibodies to be counterstained with DAPI (Thermo Fisher) and mounted in FluorSave (Merck Millipore) later. The following primary antibodies were used at the indicated dilutions: goat anti-Pannexin1 (catalog SC49695, Santa Cruz Biotechnology, 1:50), mouse anti-NeuN 1:100, mouse anti-GFAP 1:200 (Sigma-Aldrich). Donkey anti-mouse or anti-goat secondary antibodies, conjugated with Alexa 488 or 647 (Life Technologies, Madrid, Spain) were used at 1:500. Confocal images were acquired with a TCS SPE Confocal microscope equipped with four laser lines (405, 488, 561, and 653 nm) using a X63 oil objective (Leica Microsystems, Wetzlar, Germany) using the Leica software LAS AF v2.2.1 software (Leica Microsystems).

### Statistical Analysis

Data are shown as mean values ± SEM. The numbers of mice per group or genotype used in each experiment are annotated in the corresponding figure legends as n. All experiments shown were reproduced 3–5 times independently. Figures and statistical analyses were generated using GraphPad Prism 6 (GraphPad Software). Results were analyzed by un-paired Student’s *t*-tests. The statistical test used and *p*-values are indicated in each figure legend. *p* < 0.05 was considered statistically significant. ^∗^*p* < 0.05, ^∗∗^*p* < 0.01, and ns, not significant.

## Results

### TNAP+/- Brain Shows Reduced Alkaline Phosphatase Activity

**FIGURE 1:**
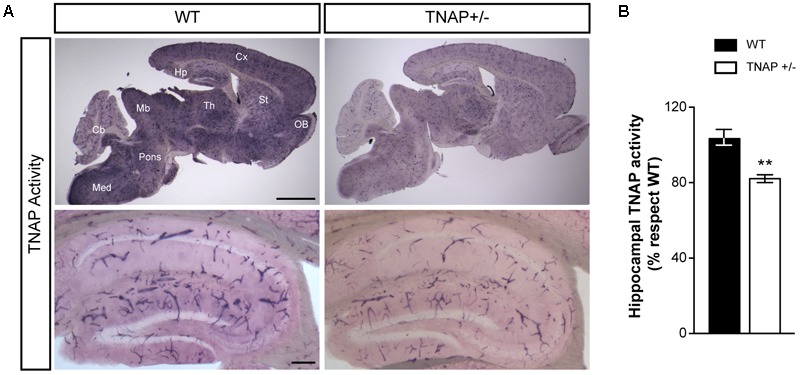
TNAP+/– brain presents reduced alkaline phosphatase activity. **(A)** TNAP enzymatic assays on brain slices shows an overall (upper panels) and a hippocampal (lower panels) decrease of alkaline phosphatase activity in TNAP+/– mice compared with WT. Cx, neocortex; Hp, hippocampus; Th, thalamus; St, striatum; OB, olfactory bulbs; Mb, midbrain; Cb, cerebellum; Med, medulla. Scale bars: 2 mm (upper panels) and 200 μm (lower panels). **(B)** Graph shows quantification of the hippocampal TNAP activity (*n* = 6 mice per genotype; sections = 3 per mouse). The 100% value corresponds to the BCIP/NBT precipitated amount detected in the hippocampus of WT mice. Data are given as means ± SEM, ^∗∗^*p* < 0.01, using unpaired Student’s *t*-test.

We first sought evidence that the decreased genetic load of TNAP in heterozygous mice is associated with lower cerebral TNAP activity. Therefore, we performed TNAP enzymatic assays in parasagittal brain slices using WT and TNAP+/- mice. We observed high levels of TNAP activity in most structures of the adult WT brain including the neocortex, hippocampus and thalamus among others, except in the cerebellum. As expected, all these structures displayed lower alkaline phosphatase activity in TNAP+/- mice than in their corresponding WT littermates (**Figure 1A**, upper panels). As previously described, images at higher magnifications also revealed, in both genotypes, a high presence of catalytic activity associated with blood vessels of various calibers (Vorbrodt et al., 1986; Langer et al., 2008). Focusing on the hippocampus, we observed a significant decreased activity in the microvessels from TNAP+/- mice, and even clearer in their parenchyma (**Figure 1A**, lower panels and **Figure 1B**). These findings confirm TNAP+/- mice are a hypomorphic model for TNAP activity in the brain.

### Increased Susceptibility of TNAP+/- Mice to ATP-Induced Electrographic Seizures

**FIGURE 2:**
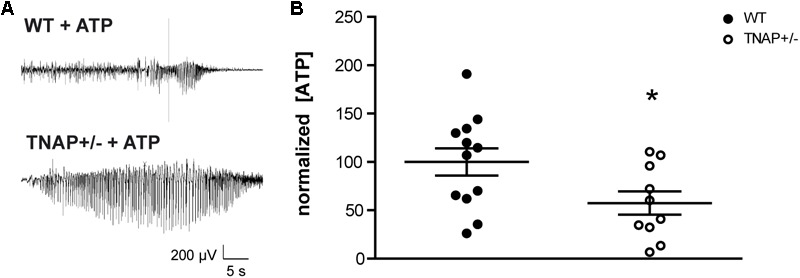
TNAP+/– CSF present lower levels of ATP. **(A)** Representative example of EEG trace recorded showing high frequency high amplitude spiking during ATP-induced seizures in WT and TNAP+/– mice. **(B)** ATP concentrations were measured in CSF samples collected from mice (*n* = 12 WT and *n* = 10 TNAP+/–) using a commercially available bioluminescence kit. The 100% value corresponds to 6.63 ± 1.78 pmol/μL of ATP detected in the CSF. Data are given as means ± SEM, ^∗^*p* < 0.05, using unpaired Student’s *t*-test.

Recent work from our group has demonstrated that the i.c.v. administration of ATP induces seizures in adult WT and TNAP+/- mice by activating P2X7R. Accordingly, both the pharmacological blockage and the genetic depletion of P2X7R prevented ATP-induced seizures (Sebastián-Serrano et al., 2016). Here, we sought to extend these earlier findings by testing directly the response of TNAP+/- mice to an intracerebral injection of ATP to mimic a pathologic event such as cell death or prolonged seizure activity. We found that the i.c.v. administration of 2 μl of 0.5 M ATP induced a more prolonged and intense single seizure in TNAP+/- than in WT mice (**Figure 2A**). The average time of convulsive seizures was 14.2 ± 8.1 s in WT mice (*n* = 7) and 81.0 ± 41.9 s in TNAP+/- mice (*n* = 5) (*p* < 0.05), similar as previously reported (Sebastián-Serrano et al., 2016). The analysis of the mean power frequency of ATP-evoked seizures revealed that although slightly higher in WT mice, there was no significant difference between both groups (WT = 9.92 ± 1.5 Hz and TNAP 12.95 ± 4.01 Hz, *p* = 0.47, by Student’s two-tailed *t*-test *n* = 3 WT and 5 TNAP+/- mice).

### Decreased ATP Concentration in the CSF of TNAP+/- Mice

Despite their high susceptibility to ATP-induced seizures, TNAP+/- mice do not display spontaneous seizures (Waymire et al., 1995; Narisawa et al., 1997). In order to evaluate if TNAP+/- mice develop any compensatory mechanisms suppressing the development of spontaneous seizures, we decided to analyze the extracellular levels of ATP. To this end, we collected samples of the CSF from the cisterna magna of WT and TNAP+/- mice. Extracellular ATP levels were measured using rLuciferase/Luciferin reagent. Surprisingly, we found ATP levels were significantly lower in TNAP+/- mice when compared WT mice (57.41 ± 12% relative to WT) (**Figure 2B**).

### Normal Physiological Cell Death in TNAP+/- Mice

**FIGURE 3:**
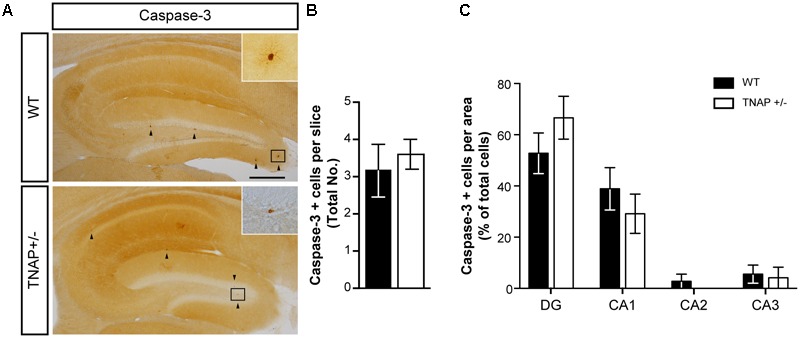
Unaltered physiological cell death in TNAP+/– mice. **(A)** Image showing caspase-3 immunoreactivity in the hippocampus of WT and TNAP+/– mice. Inset shows detail of caspase-3 positive cells. Black arrowheads indicate samples of caspase-3 positive cells. Scale bar: 200 μm. **(B)** Total number of caspase-3 positive cells and **(C)** their distribution throughout the hippocampus (*n* = 6 mice per genotype; sections = 3 per mouse). Data are given as means ± SEM.

Because under pathological conditions cell death is an important source responsible for large increases in extracellular ATP, we decided to analyze whether decreased extracellular ATP levels in TNAP +/- brains was associated with lower cellular mortality. To evaluate this point, we performed immunohistochemical assays against caspase-3, an indicator of apoptosis. Occasionally, we found a few positive cells with active caspase-3 in the hippocampus of both genotypes (**Figure 3A**), however, we did not detect any significant differences both in the total number (**Figure 3B**) or distribution (**Figure 3C**) of caspase-3 positive cells between both genotypes. Similar results were observed in cerebral cortex (Supplementary Figure 1).

### TNAP+/- Mice Present Reduced Hippocampal Ecto-ATPase Activity

**FIGURE 4:**
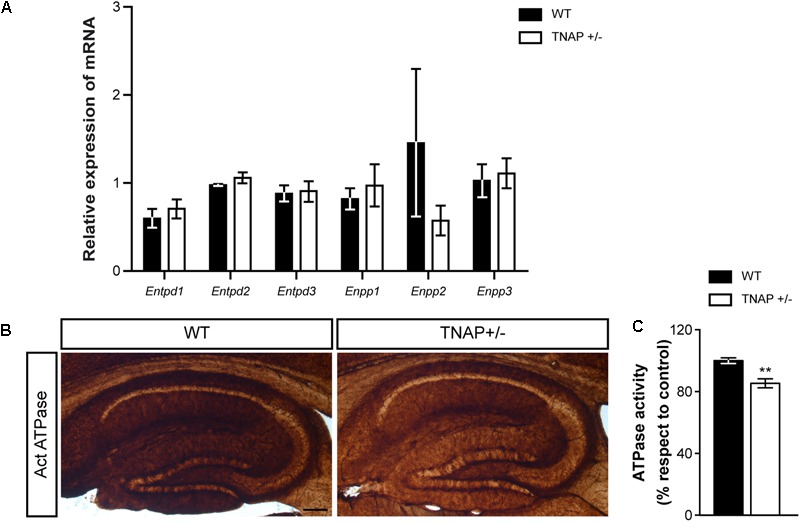
TNAP+/– brain presents diminished ecto-ATPase activity. **(A)** Lack of upregulation of other CNS ectonucleotidases in TNAP+/– mice as revealed by qRT-PCR. The graph represents quantification of the relative abundance of *Entpd1, Entpd2, Entpd3, Enpp1, Enpp2*, and *Enpp3* mRNA from the hippocampus of WT and TNAP+/– mice (*n* = 6 per genotype). **(B)** Enzyme histochemical reaction products in parasagittal sections using ATP as a substrate for visualizing ecto-ATPase activity. Images show a more intense staining in the hippocampus of WT mice when compared to TNAP+/–. Scale bar: 200 μm. **(C)** Graph shows quantification of the hippocampal ATPase activity (*n* = 4 mice per genotype; sections = 3 per mouse). The 100% value corresponds to the ATPase activity detected in the hippocampus of WT mice. Data are given as means ± SEM, ^∗∗^*p* < 0.01, using unpaired Student’s *t*-test.

Next, we wondered whether the decreased alkaline phosphatase activity observed in TNAP heterozygous mice was compensated by changes in the expression of other ectonucleotidases. To answer this question, we measured messenger RNA levels of the main CNS ectonucleotidases capable of hydrolyzing extracellular nucleotide triphosphates. Specifically, we focused on surface-located members of E-NTPDase and E-NPP families encoded respectively by the genes *Entpd* and *Enpp* (Zimmermann, 2006). The results showed that WT and TNAP+/- mice present similar mRNA levels of the different E-NTPDases and E-NPPs analyzed (**Figure 4A**). Accordingly, the lower ecto-ATPase activity does not appear to prompt compensatory gene expression responses in other nucleotidases (**Figures 4B,C**).

### Reduced Panx1 Protein Levels in the Brain of TNAP+/- Mice

**FIGURE 5:**
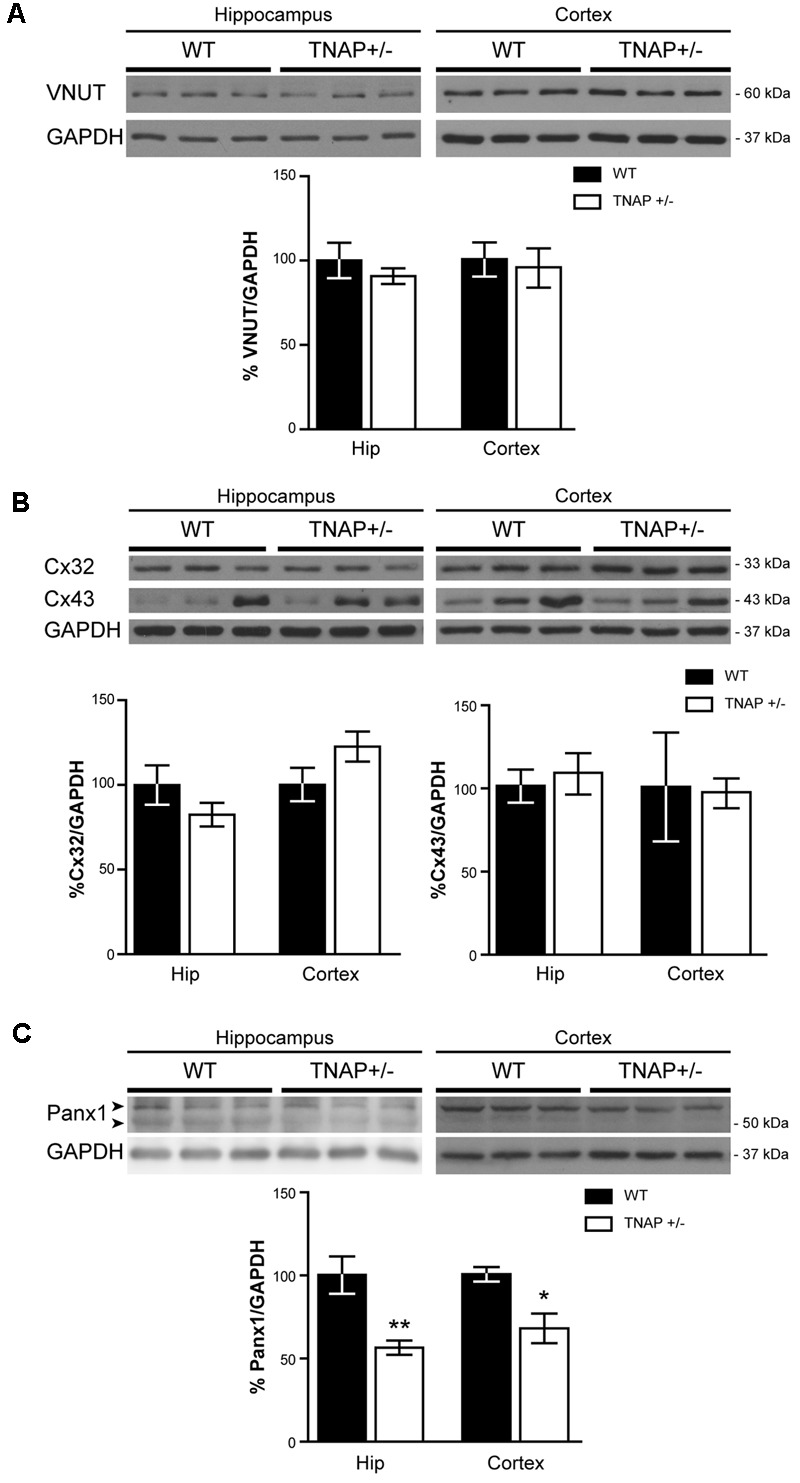
Panx1 is downregulated in TNAP+/– hippocampus. Representative Western blot and quantification of the protein expression of **(A)** VNUT, **(B)**, connexin 32 (Cx32) and connexin 43 (Cx43) and **(C)** Panx1 (*n* = 4 WT and *n* = 5 TNAP+/–). Data are normalized to the expression levels of GAPDH “housekeeping” gene. Data are given as means ± SEM, ^∗^*p* < 0.05 and ^∗∗^*p* < 0.01, using unpaired Student’s *t*-test.

Next, to determinate if the observed reduction in ATP levels was related with an altered exocytotic release of this nucleotide, we decided to measure protein levels of VNUT responsible for the storage of ATP in secretory vesicles (Sawada et al., 2008). Lysates from hippocampal and cortical samples were subjected to immunoblotting assays using specific antibodies against VNUT protein. Western blot analysis using the polyclonal antibody anti-VNUT PA5-63312 from Thermo Fisher showed that VNUT protein levels were similar between WT and TNAP+/- mice both in the hippocampus and cerebral cortex (**Figure 5A**). Similar results were obtained using the polyclonal antibody against VNUT ABN110 from Millipore (Supplementary Figure 2).

We next considered changes to non-exocytotic mechanisms in the TNAP+/- mice. Neural cells, in response to a variety of different stimuli, are also capable of releasing ATP by non-secretory mechanisms involving mainly membrane channels. To explore if the decrease in ATP levels detected in TNAP+/- mice is the result of reduced expression of these channels we measured the levels of connexin 32 and 43 hemichannels, the principal connexins involved in the ATP release in oligodendrocytes and astrocytes respectively (Nagy and Rash, 2000). Hippocampal expression of both connexins was similar between TNAP+/- and WT mice (**Figure 5B**). Similar results were observed in the cerebral cortex (**Figure 5B**).

**FIGURE 6:**
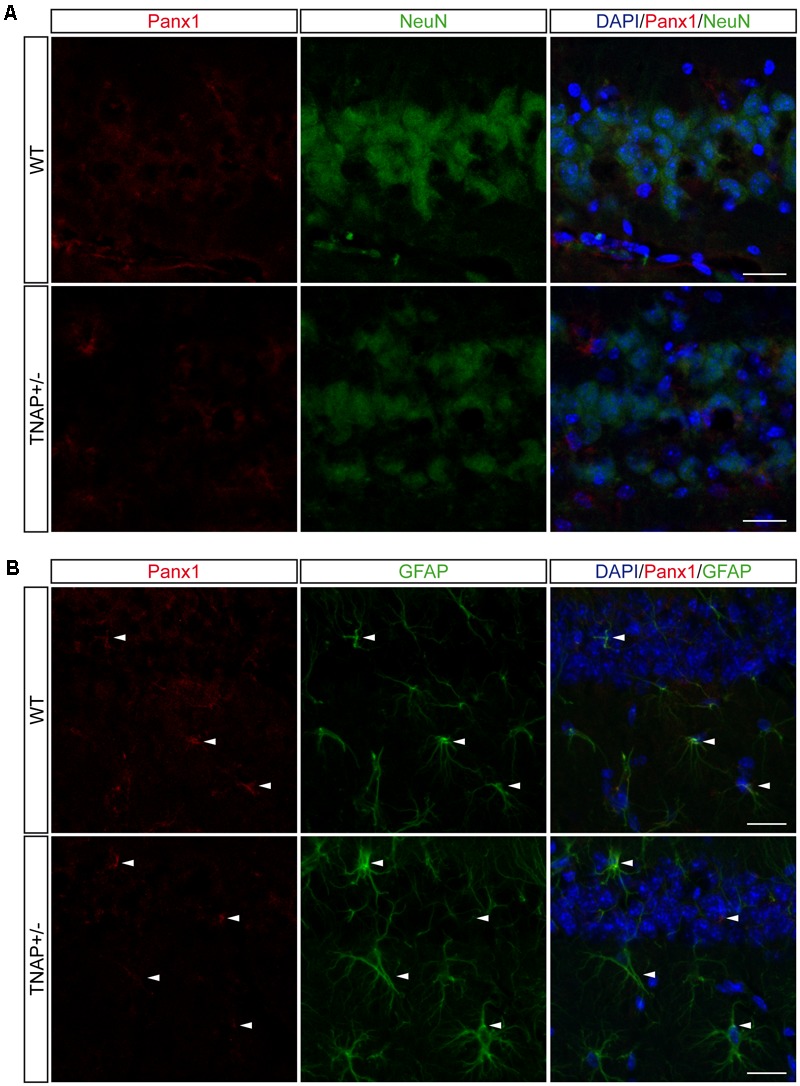
Location of Panx1 in the hippocampus. Representative images of hippocampal parasagittal sections from TNAP+/– and WT mice co-stained with an antibody against pannexin-1 (red channel) and **(A)** neuronal marker NeuN (green channel) or **(B)** astroglial marker GFAP (green channel) and counterstained with nuclear marker DAPI. White arrowheads point to double stained astrocytes. Scale bar: 50 μm.

Finally, because Panx1 is a protein forming hemichannels that can release extracellular ATP in a non-secretory manner (Huang et al., 2007; Chekeni et al., 2010), we decided to explore Panx1 levels in both genotypes. It should be noted that Panx1 is an extensively glycosylated protein, showing multiple specific bands when performing western blotting (Supplementary Figure 4) (Penuela et al., 2007). Hippocampal and cortical Panx1 levels were lower in TNAP+/- mice than in their corresponding WT littermates (**Figure 5C**). Double immunofluorescence using specific neuronal (NeuN) and astroglial (GFAP) markers revealed that a reduction in Panx1 expression took mainly place in neurons (**Figures 6A,B**). This finding suggests there is a select deficit in the non-secretory ATP release pathway that may underlie the lower extracellular ATP concentration found in TNAP+/- mice. Because in previous work we reported that TNAP-/- mice presented higher extracellular ATP levels in the CNS than their WT littermates (Sebastián-Serrano et al., 2016), we decided to measure their Panx1 levels. Since TNAP-/- mice died around the postnatal day 10, we measured the Panx1 levels in cortical samples obtained from neonatal TNAP-/- and TNAP+/- mice at postnatal day 9. Unexpectedly, results revealed that both TNAP-/- and TNAP+/- mice showed similar Panx1 levels than those observed in their WT littermates (Supplementary Figure 3).

## Discussion

In the current study, we confirm that the brains from adult TNAP+/- mice show an overall decrease in alkaline phosphatase activity. Unexpectedly, these mice displayed lower extracellular ATP levels in the CSF than those detected in their corresponding WT littermates. This decrease was neither compensated by changes in the expression or increased activity of other ectonucleotidases nor by a decreased expression of VNUT, which could have down-regulated the exocytotic ATP release. In addition, no alterations in cell death was detected between genotypes. Regarding the proteins related to the non-exocytotic release of ATP, we did not find alterations in the expression levels of the main connexins expressed in glial cells (Nagy and Rash, 2000). However, a significant decrease in the protein levels of Panx1 was observed in TNAP+/- mice. Because Panx1, a single-membrane, large-conductance channel (Sosinsky et al., 2011) has been widely related to the non-exocytotic ATP release (Heinrich et al., 2012; Prochnow et al., 2012; Wicki-Stordeur et al., 2012), our results suggest that this adjustment may be a compensative mechanism of adult TNAP+/- mice to regulate the brain extracellular ATP levels (Engel et al., 2012; Sebastián-Serrano et al., 2015, 2016). Nevertheless, because other channels have also been related with the non-vesicular ATP release from astrocytes, such as maxi-chloride channels (Liu et al., 2008), additional studies are necessary to evaluate their possible contribution in the regulation of extracellular ATP levels in TNAP+/- mice.

Over the past years, several lines of evidence have suggested that Panx1 plays a key role in cellular epileptogenesis. For example, increased expression of this channel protein has been found in surgically resected cortex of patients with temporal lobe epilepsy (Jiang et al., 2013). Notably, enhanced Panx1 mRNA expression is positively correlated with seizure frequency (Li et al., 2017). Moreover, areas with high Panx1 expression are regions that become hyperexcitable in experimental models of epilepsy (Leung and Wu, 2006). These results strongly suggest that this protein is involved in the generation of epileptic events. Studies have also supported anticonvulsant functions. Silencing expression of Panx1 was reported to increase susceptibility to seizures induced by muscarinic acetylcholine receptor activation (Kim and Kang, 2011). In addition, data also showed that the ingestion of the Panx1 blocker Mefloquine resulted in convulsions in humans with a prior history of seizures (Bem et al., 1992). Nevertheless, other groups suggested a pro-seizure role of this channel based on the anticonvulsive consequences of its pharmacological blockade. Pharmacological inhibition of Panx1 decreased the spiking amplitude of the epileptiform burst activity induced by activating NMDA receptors under Mg^2+^-free conditions in the hippocampus (Thompson et al., 2008) and prevented both the ATP release and the neuronal oscillations induced by the activation of metabotropic glutamate receptors in hippocampus (Lopatar et al., 2015) and cerebral cortex (Cepeda et al., 2015). In addition, genetic depletion of Panx1 results in the abolition of epileptiform behavior in mice exposed to kainic acid (Santiago et al., 2011). Although the reduction of Panx1 expression observed in TNAP+/- mice is not necessarily associated with a loss of function, its downregulation via interference RNA or its pharmacological blockage might contribute to regulate the basal ATP levels decreasing the non-vesicular extracellular ATP release.

In previous work, we have demonstrated that ATP-induced seizures are mediated through the P2X7R activation (Engel et al., 2012; Sebastián-Serrano et al., 2016). We established that the TNAP-/- mice reduce, at early ages, their neuronal P2X7R levels which may be a compensatory mechanism to counteract the development of spontaneous seizures induced by the increased extracellular ATP levels in the absence of this ectoenzyme (Sebastián-Serrano et al., 2016). In the present study, we found that adult TNAP+/- mice show a significant decrease of the TNAP function resulting in a reduced ecto-ATPase activity in the brain. Interestingly, although they show similar P2X7R levels than their WT littermates, do not develop spontaneous seizures and are more susceptible to ATP-induced seizures than WT mice (Sebastián-Serrano et al., 2016). Because we found that TNAP+/- mice have lower ATP levels in the CSF than their WT littermates, we reasoned that, besides P2X7R, factors regulating extracellular ATP levels might take part in the molecular mechanisms underlying the seizures associated with HPP. The analysis of the main elements involved in ATP release revealed that TNAP+/- mice only have significantly reduced the expression of Panx1. Altogether, these data suggest that alterations in TNAP activity may be compensated by different molecular mechanisms depending on its severity. So, with a partial decrease of TNAP activity, the alteration in the ATP basal levels caused by deficient ATP clearance may be balanced by decreasing the Panx1 levels. Where there is a complete lack of TNAP activity, the subsequent increase in the extracellular ATP levels induces spontaneous seizures and requires more extensive compensatory mechanisms with greater adverse outcomes as changes to P2X7R levels. Based on this hypothesis, we can postulate that although TNAP+/- and WT mice show similar expression of P2X7R, the low ATPase activity of TNAP+/- mice would contribute to extend the half-life of extracellular ATP. This event might contribute to increase the susceptibility to seizures induced by the addition of exogenous ATP. Therefore, it is reasonable to think that during growth, TNAP+/- mice reduce the expression of the Panx1 as a compensatory mechanism to reduce the basal levels of extracellular ATP, which would contribute to avoiding the seizures associated. In addition, it has been described that during the brain maturation both P2X7R and Panx1 undergo changes in location and function (Prochnow et al., 2012; Miras-Portugal et al., 2017), so we cannot discard an age-dependent component. Supporting the presence of an age-dependent factor involved in the molecular mechanism underlying the down-regulation of Panx1 neither TNAP-/- nor TNAP+/- mice showed a reduction of Panx1 levels at early ages. So, the short life expectancy of TNAP-/- mice would avoid they may develop the compensative down-regulation of Panx1. However, additional studies should be done to elucidate this hypothesis.

In summary, here we demonstrate that adult TNAP+/- mice present reduced alkaline phosphatase activity in the brain. This decrease correlates with apparent compensatory changes in CSF extracellular ATP levels, at least in part, by diminution in Panx1 protein. The lack of spontaneous seizures in TNAP+/- mice point to the molecular mechanisms underlying the release of extracellular ATP as a new avenue for the therapeutic intervention of HPP-related seizures.

## Author Contributions

ÁS-S prepared protein extract, realized Western blot, immunohistochemistry, TNAP activity test, RT-qPCRs, ATP measurement, participated in experimental design, in the interpretation of the work and wrote the manuscript. LdD-G performed Western blot of hemichannels, ecto-ATPase activity assay, and revised the manuscript. TE performed *in vivo* seizure induction and recording and revised the manuscript. DH revised the manuscript. MD-H performed CSF acquisition and ATP measurement, participated in the experimental design, in the interpretation of the results, wrote the manuscript and also provided the financial support for the work.

## Conflict of Interest Statement

The authors declare that the research was conducted in the absence of any commercial or financial relationships that could be construed as a potential conflict of interest.

## Abbreviations

ATP: adenosine 5′-triphosphate
BSA: bovine serum albumin
CNS: central nervous system
CSF: cerebrospinal fluid
DAB: 3, 3′-diaminobenzidine
DAPI: 4′,6-diamidine-2′-phenylindole dihydrochloride
EEG: electroencephalogram
FBS: fetal bovine serum
GABA: γ-aminobutyric acid
HPP: hypophosphatasia
i.c.v.: intracerebroventricular
M-MLV: Moloney murine leukemia virus
Panx1: pannexin-1
PBS: phosphate-buffered saline
PBS-T: PBS-buffer containing 0.1% (v/v) Tween-20
PFA: 4% paraformaldehyde in PBS
P2X7R: P2X7 receptor
qRT-PCR: quantitative real-time PCR
S.E.M.: standard error of the mean
TMS: Tris-maleate sucrose buffer
TNAP: tissue-nonspecific alkaline phosphatase
TNAP-/-: *Akp2* knockout mice
TNAP+/-: heterozygous TNAP mice
VNUT: vesicular nucleotide transporter
WT: wild type
