# Supplementary material for: Haploinsufficient TNAP Mice Display Decreased Extracellular ATP Levels and Expression of Pannexin-1 Channels
Source: Front Pharmacol. 2018 Mar 2;9:170. doi: 10.3389/fphar.2018.00170 (PMC5841270; doi:10.3389/fphar.2018.00170)
Supplement: Supplementary file 1 [file Data_Sheet_1.pdf]

Supplementary Material

Happloinsufficient TNAP Mice Display Decreased Extracellular ATP Levels and Expression of Pannexin-1 Channels

Álvaro Sebastián-Serrano, Laura de Diego-García, David C. Henshall, Tobías Engel and Miguel Díaz-Hernández *

*** Correspondence:** Professor Miguel Díaz-Hernández
migueldiaz@ucm.es

Supplementary Figures.

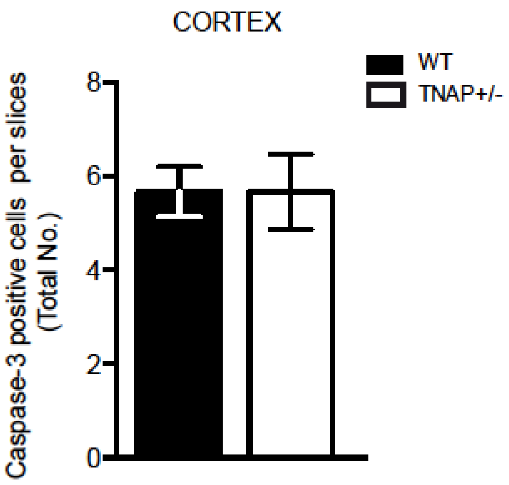

SUPPLEMENTARY FIGURE 1. Unaltered physiological cell death in TNAP+/- mice. Graph represents the total number of caspase-3 positive cells in neocortex from WT and TNAP +/- mice (n = 6 mice per genotype; sections = 3 per mouse). Data are given as means ± s.e.m.

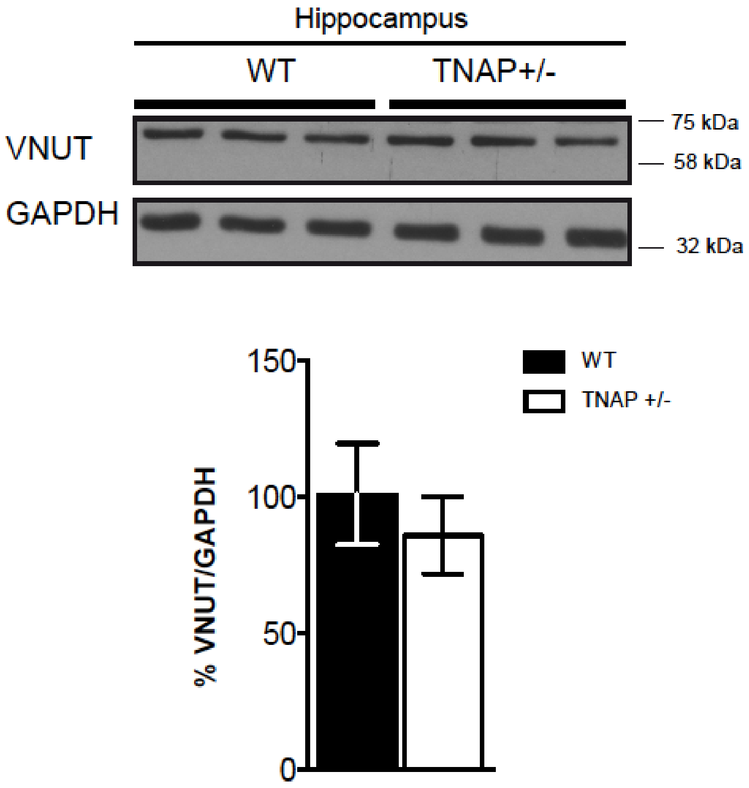

SUPPLEMENTARY FIGURE 2. Detection of VNUT levels in hippocampus from TNAP+/- and WT mice. Representative Western blot and quantification of the protein expression of VNUT using ABN110 antibody from millipore (n = 4 WT, n = 5 TNAP+/-). Data are normalized to the expression levels of GAPDH "housekeeping" gene. Data are given as means ± s.e.m.

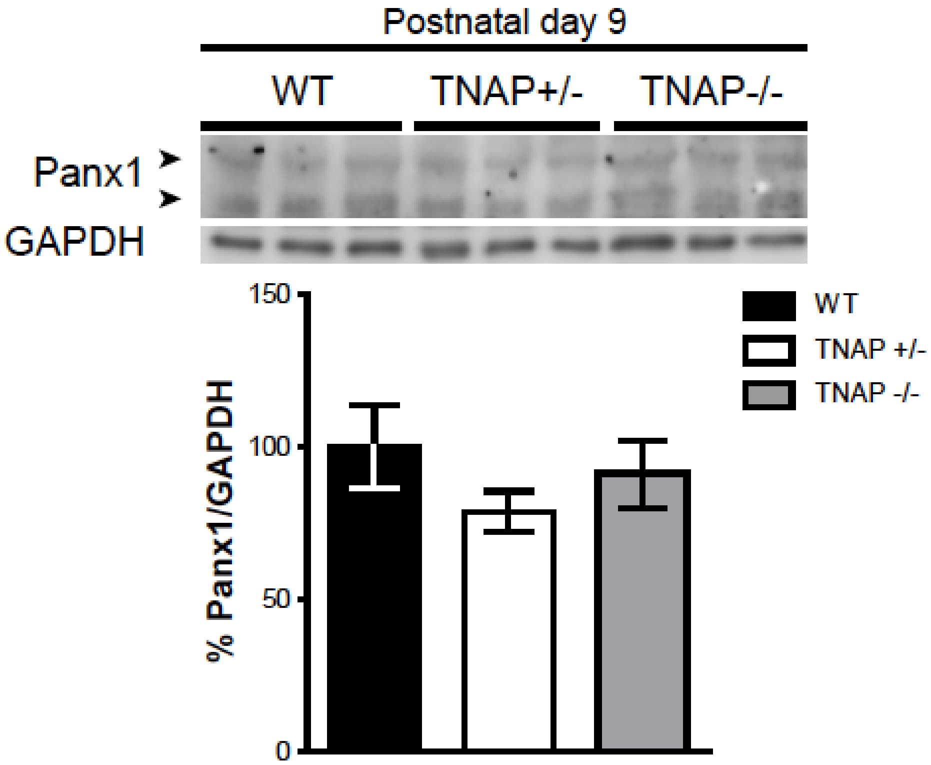

SUPPLEMENTARY FIGURE 3. Panx1 do not change in brain from either TNAP+/- or TNAP-/- postnatal mice. Representative Western blot and quantification of the protein expression of Panx1 (n = 3 WT, n = 3 TNAP+/- and n = 3 TNAP-/-). Data are normalized to the expression levels of GAPDH "housekeeping" gene. Data are given as means ± s.e.m.

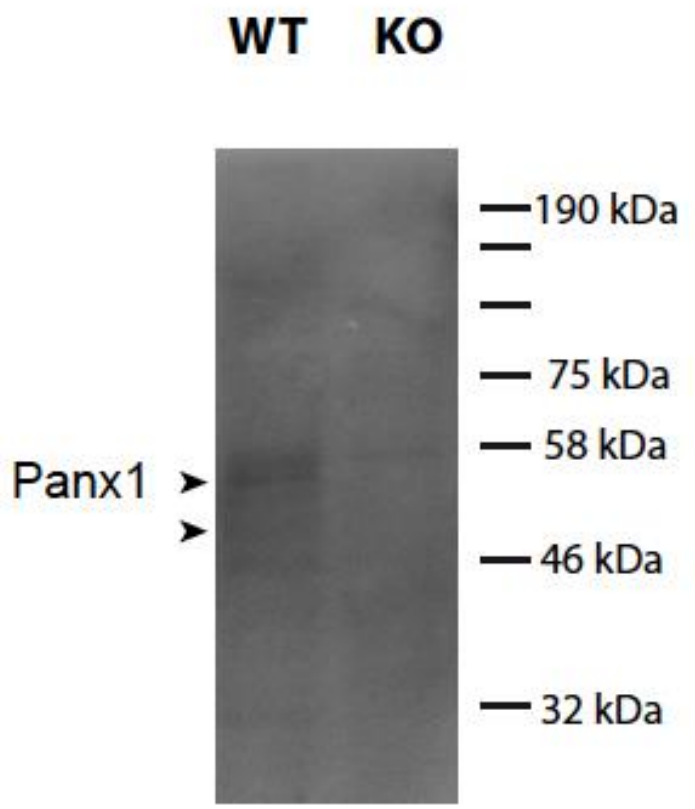

SUPPLEMENTARY FIGURE 4. Detection of Panx1 protein in spleen samples from WT and Panx1-/-mice. Representative Western blot using spleen samples from WT and Panx1-/- mice stained with anti-Panx1 antibody SC49695 from Santa Cruz. Specific bands analysed are indicated by arrowheads. Spleen samples were kindly gift by Prof. Pablo Pelegrín and Dr. Gonzalo de la Rosa.

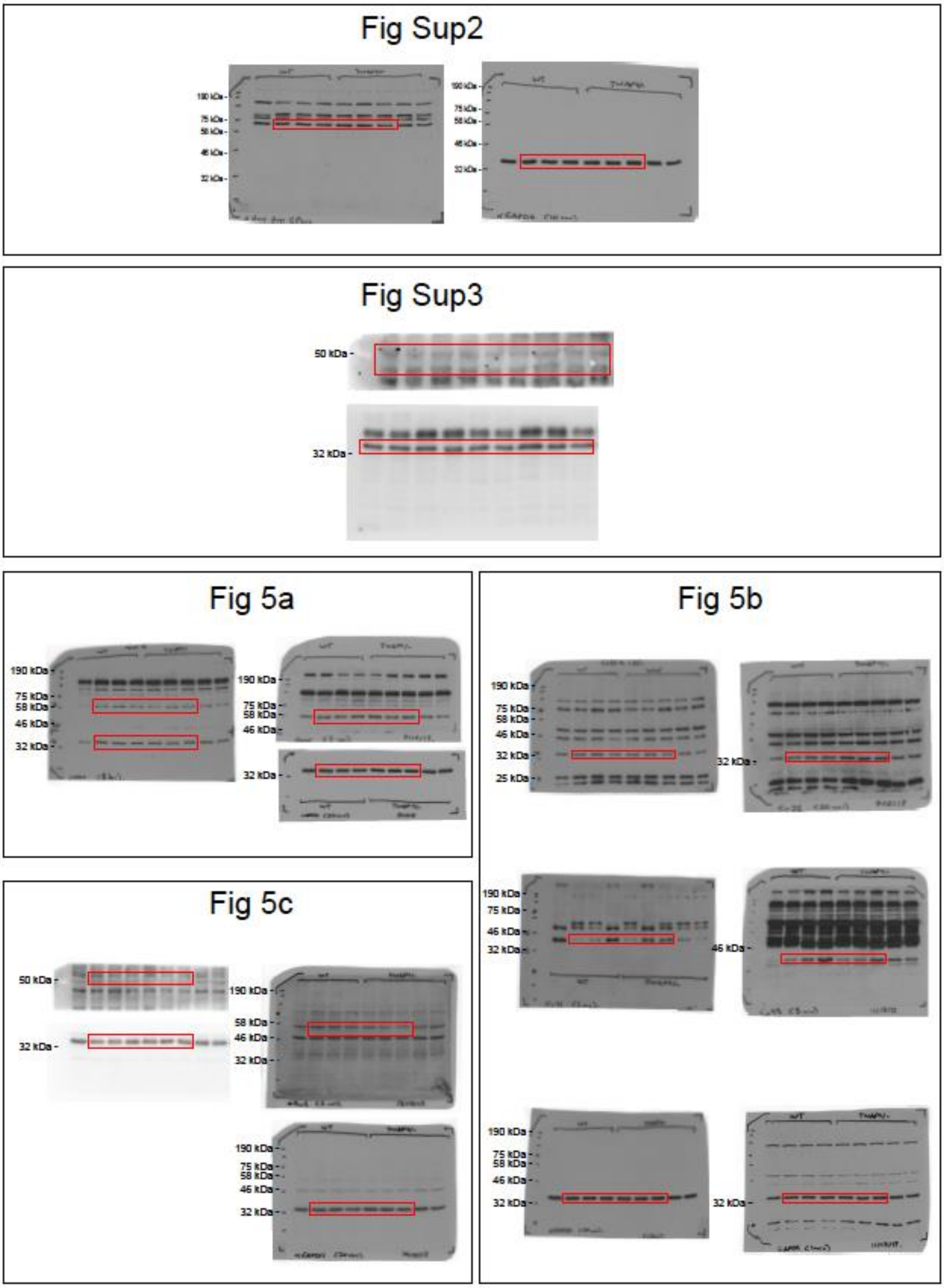

SUPPLEMENTARY FIGURE 5. Complete Western blots for Figure 5a, Figure 5b, Figure 5c and Supplementary Figure 2 and 3.
